# Validation of an epigenetic field of susceptibility to detect significant prostate cancer from non-tumor biopsies

**DOI:** 10.1186/s13148-019-0771-5

**Published:** 2019-11-28

**Authors:** Bing Yang, Tyler Etheridge, Johnathon McCormick, Adam Schultz, Tariq A. Khemees, Nathan Damaschke, Glen Leverson, Kaitlin Woo, Geoffrey A. Sonn, Eric A. Klein, Mike Fumo, Wei Huang, David F. Jarrard

**Affiliations:** 1 University of Wisconsin School of Medicine and Public Health, Madison, WI 53705 USA; 2 Department of Urology, University of Wisconsin, Madison, WI 53705 USA; 3 Stanford University School of Medicine, Stanford, CA USA; 4 Cleveland Clinic, Glickman Urological and Kidney Institute, Cleveland, OH USA; 5 Rockford Urologic, Rockford, IL USA; 6 Carbone Comprehensive Cancer Center, University of Wisconsin, Madison, WI 53705 USA; 7 Molecular and Environmental Toxicology Program, University of Wisconsin, Madison, WI 53705 USA

**Keywords:** Prostate cancer, Epigenetics, DNA methylation, Field defect

## Abstract

**Background:**

An epigenetic field of cancer susceptibility exists for prostate cancer (PC) that gives rise to multifocal disease in the peripheral prostate. In previous work, genome-wide DNA methylation profiling identified altered regions in the normal prostate tissue of men with PC. In the current multicenter study, we examined the predictive strength of a panel of loci to detect cancer presence and grade in patients with negative biopsy tissue.

**Results:**

Four centers contributed benign prostate biopsy tissues blocks from 129 subjects that were either tumor associated (TA, Grade Group [GG] ≥ 2, *n* = 77) or non-tumor associated (NTA, *n* = 52). Biopsies were analyzed using pyrosequencing for DNA methylation encompassing CpG loci near *CAV1*, *EVX1*, *FGF1*, *NCR2*, *PLA2G16*, and *SPAG4* and methylation differences were detected within all gene regions (*p* < 0.05). A multiplex regression model for biomarker performance incorporating a gene combination discriminated TA from NTA tissues (area under the curve [AUC] 0.747, *p* = 0.004). A multiplex model incorporating all the above genes and clinical information (PSA, age) identified patients with GG ≥ 2 PC (AUC 0.815, *p* < 0.0001). In patients with cancer, increased variation in gene methylation levels occurs between biopsies across the prostate.

**Conclusions:**

A widespread epigenetic field defect is utilized to detect GG ≥ 2 PC in patients with histologically negative biopsies. These alterations in non-tumor cells display increased heterogeneity of methylation extent and are spatially distant from tumor foci. These findings have the potential to decrease the need for repeated prostate biopsy.

## Background

Prostate cancer (PC) is the most frequently observed cancer in men, with approximately 1 in 6 diagnosed in their lifetime [1]. Despite its high incidence, PC detection remains clinically challenging. Typically, prostate-specific antigen (PSA) is used to detect PC, and if abnormal, a 10–12 core biopsy is obtained under ultrasound guidance. Over 40% of patients with a negative biopsy receive a second biopsy, and many will receive additional biopsies in an effort to detect this microscopic disease [2]. Indeed, repeat biopsies account for roughly 780,000 of the 1.2 million biopsies done annually. MRI has improved the detection of larger volume cancers, but roughly 30% of significant PCs remain undetected by this approach [3]. The biology of this common, multifocal, and microscopic disease presents unique genomic opportunities to improve its detection.

The concept of a field defect, which can explain the multifocality of some cancers, including prostate, colon, and bladder [4–6], suggests that preneoplastic molecular alterations may exist in benign tissues [2]. The predilection of PC for the peripheral zone of the prostate and its frequent multifocality suggests a field of susceptibility. This field change strongly links to epigenetic alterations, the initial finding being a loss of genomic imprinting for the insulin-like growth factor-2 (IGF2) gene [7, 8]. It is also characterized by a panel of DNA methylation changes at specific loci that persist even in regions spatially remote (over 1 cm) from tumor-bearing areas [4]. Because of the widespread nature of these methylation changes in normal tissue, their use may offer increased sensitivity over diagnostic approaches using methylation associated with peritumor or “halo” alterations found in some benign tissues adjacent to cancer [2].

This field of susceptibility offers an opportunity for improved detection of the disease. The primary objective of this study was to further define these methylation patterns in tissue biopsies and validate a panel of methylated regions as a method for detecting higher risk PC in men with histologically negative biopsies.

## Results

### Patient characteristics

Table 1 displays clinical data for the study cohorts. Benign biopsy cores were obtained from two cohorts: (1) Tumor-associated (TA) patients diagnosed with GG ≥ 2 cancer (*n* = 77), and (2) non-tumor-associated (NTA) patients (*n* = 52) who had no cancer on any biopsy core. None of the analyzed biopsy cores from either cohort contained cancer. All TA patients also had a radical prostatectomy to confirm a final pathologic Grade Group (GG ≥ 2) consistent with the study’s goal of focusing on the detection of clinically significant PC. TA and NTA cohorts are matched for age and PSA density. PSA (7 vs 5.8; *p* < 0.01) and prostate size (47 g vs 36 g; *p* < 0.01) are increased in the NTA group compared with the TA group demonstrating the limited potential of PSA in detecting cancer in this population.

**Table 1:** Clinicopathological features of non-tumor-associated (NTA) and tumor-associated (TA) study groups

	NTA	TA	Total	*p* value
Patients, *n*	52	77	129	---
Cleveland Clinic	9	25	34	---
Rockford Clinic	20	19	39	---
Stanford Univ.	3	6	9	---
UW-Madison	20	27	47	---
Age (year)	60.3 (50–70)	61.3 (51–70)	60.9 (50–70)	0.22
PSA (ng/mL)*	7.0 (3.3–15.0)	5.8 (2.4–10.6)	6.3 (2.4–15.0)	< 0.01
PSA density (ng/mL)*	0.172 (0.06–0.43)	0.173 (0.06–0.40)	0.174 (0.06–0.43)	0.89
Prostate size (g)	46.6 (20–150)	36.3 (15–70)	40.3 (15–150)	< 0.01
BMI (kg/m^2^)*	29.69 (21.2–51.2)	29.11 (20.9–41.0)	29.34 (20.9–51.2)	0.69
Ethnicity:				---
Caucasian	94.2% (49/52)	88.3% (68/77)	90.7% (117/129)	---
Family History:*				---
Positive	25.0% (12/48)	35.6% (26/73)	31.4% (38/121)	---
DRE:*				---
Positive	13.7% (7/51)	13.3% (10/75)	13.5% (17/126)	---
Grade Group (Gleason Score):				---
2 (3 + 4)	---	36	36	---
3 (4 + 3)	---	29	29	---
4 (4 + 4)	---	4	4	---
5 (4 + 5, 5 + 4)	---	8	8	---
Pathological stage:				---
T2a	---	6	6	---
T2b	---	9	9	---
T2c	---	39	39	---
T3a	---	18	18	---
T3b	---	5	5	---

*Some samples are missing data; *TA*, tumor associated; *NTA*, non-tumor associated; *PSA*, prostate-specific antigen; *BMI*, body mass index. All data represented as mean (range) unless otherwise specified

### Methylation assay performance for two biopsies in discriminating tumor-associated from non-tumor-associated samples

Utilizing bisulfite-treated DNA and pyrosequencing, linear results using standards are seen across the clinically pertinent methylation ranges for each of the loci tested validating this testing approach (Additional file 1: Table S1). We observe robust methylation differences between NTA and TA prostate biopsies across all regions associated with *EVX1*, *CAV1*, *PLA2G16*, and *SPAG4* (hypermethylation) and *FGF1* and *NCR2* (hypomethylation) at all CpGs assayed validating our previous exploratory studies [4, 9]. Mean, maximum, and minimum methylation levels were compared between the two biopsies for both TA and NTA tissues (Additional file 1: Tables S2–S4). Using maximal methylation values shows improved statistical significance in differentiating TA samples at hypermethylated loci, while minimal methylation values improve hypomethylated regions.

The predictive accuracy of these genes was assessed with regression models using each gene alone (uniplex) or in combination (multiplex) in Table 2. In uniplex models when examining CpGs tested, 6 out of 6 *EVX1*, 2/10 *CAV1*, 1/5 *FGF1*, 1/3 *NCR2*, 5/6 *PLA2G16*, and 2/5 *SPAG4* show strong predictive accuracies (area under the curve [AUC] 0.61–0.71, *p* < 0.05, Table 2). As a single marker, *EVX1*_CG1 generates the best AUC of 0.710, (*p* = 0.001).

**Table 2:** Uniplex and multiplex logistic regression model for biomarker performance to detect cancer using two biopsies

		Uniplex modeling			
CG	Coefficient	Constant	O.R. (95% CI)	AUC	*p* value
C-7 Max	0.0365	− 1.365	1.037 (1.004–1.072)	0.613	0.020
C10 Max	0.0666	− 1.0824	1.069 (1.005–1.137)	0.632	0.035
E-1 Max	0.0784	− 3.196	1.082 (1.035–1.130)	0.710	0.001
E-2 Max	0.0633	− 2.11	1.065 (1.023–1.110)	0.696	0.002
E-3 Max	0.0543	− 2.7005	1.056 (1.025–1.087)	0.700	0.001
E-4 Max	0.0306	− 2.3534	1.031 (1.000–1.063)	0.621	0.048
E-5 Max	0.0481	− 2.7315	1.049 (1.011–1.089)	0.692	0.011
E-6 Max	0.0575	− 1.8742	1.059 (1.012–1.109)	0.642	0.014
F-3 Min	-0.0524	3.0835	0.949 (0.908–0.992)	0.641	0.021
N-2 Min	-0.1492	5.1864	0.861 (0.755–0.982)	0.616	0.026
P-1 Max	0.0471	− 1.6977	1.048 (1.006–1.093)	0.618	0.026
P-2 Max	0.1129	− 2.1638	1.120 (1.029–1.218)	0.643	0.009
P-3 Max	0.1181	− 1.654	1.125 (1.027–1.233)	0.653	0.012
P-4 Max	0.0314	− 1.5588	1.032 (1.007–1.058)	0.642	0.014
P-5 Max	0.1119	− 2.4409	1.118 (1.036–1.208)	0.658	0.004
S-1 Max	0.0605	− 1.3402	1.062 (1.004–1.124)	0.604	0.035
S-2 Max	0.0531	− 1.5709	1.055 (1.066–1.105)	0.639	0.026
		Multiplex modeling			
CpG from each locus				0.747	0.004
C-10 Max	0.0139	0.4058	1.014 (0.906–1.135)		
E-1 Max	0.0534	0.4058	1.055 (0.998–1.115)		
F-3 Min	− 0.0182	0.4058	0.982 (0.924–1.044)		
N-2 Min	− 0.0975	0.4058	0.907 (0.785–1.048)		
P-5 Max	0.0847	0.4058	1.088 (0.945–1.253)		
S-2 Max	− 0.0242	0.4058	0.976 (0.895–1.064)		

*C*, *CAV1*; *E*, *EVX1*; *F*, *FGF1*; *N*, *NCR2*; *P*, *PLA2G16*; *S*, *SPAG4*; *Max*, maximal methylation value between the two biopsies; *Min*, minimal methylation value between the two biopsies; *O.R*., odds ratio; *AUC*, area under the curve value. Number after each letter represents the CG position tested

**Fig. 1:**
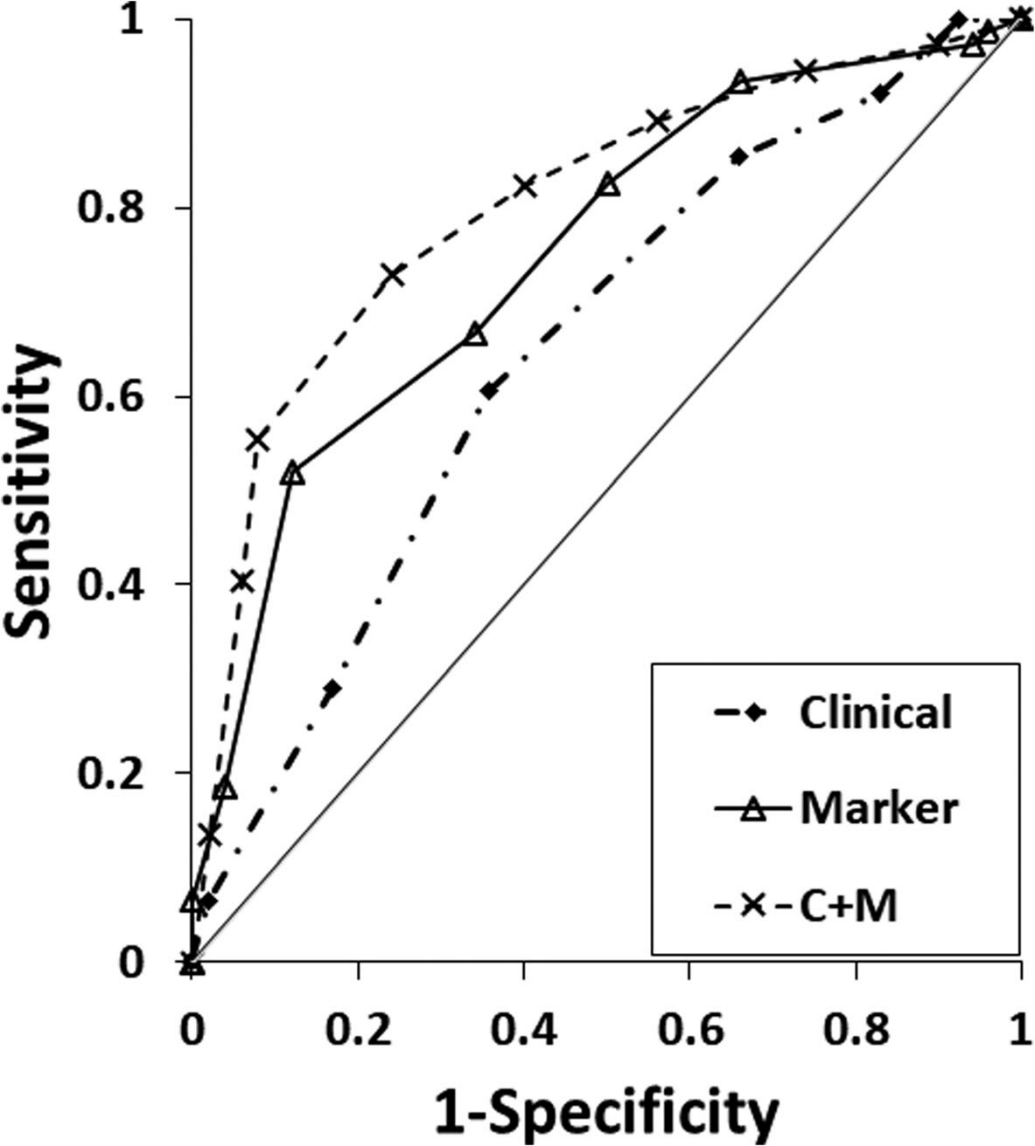
ROC for the predictive accuracy for detecting cancer using uniplex and multiplex regression models for discriminating TA and NTA biopsy negative cores (two biopsies). When pan-biomarkers used alone (Max_*CAV1* CG10, Max_*EVX1* CG1, Max_*PLA2G16* CG5, Max_*SPAG4* CG2, Min_*FGF1* CG3, and Min_*NCR2* CG2), the predictive accuracy was 0.747, *p* = 0.004 (solid curve). Clinical features (age and LogPSA) had predictive accuracy AUC 0.631, *p* = 0.005 (dashed and dotted curve). Multiplex model incorporating pan-biomarkers and clinical features (dashed curve) had highest predictive accuracy (AUC 0.815, *p* < 0.0001) for discriminating TA vs NTA biopsy negative cores

To determine whether a panel performed better than any single biomarker, we performed a multiplex analysis (Table 2). First, the collinearity of individual CpG sites using correlation matrices for every CG in each gene was assessed. Since CG sites correlated highly, only one CG with the highest predictive value (AUC) per gene was selected to enter multivariate logistic regression models to prevent overfitting. On multivariate analysis, the genes hypermethylated in TA, Max_*CAV1*-10, Max_*EVX1*-1, Max_*PLA2G16*-5, Max_*SPAG4*-2, and the genes with hypomethylated in TA, Min_*FGF1*-3, and Min_*NCR2*-2 entered the model (Table 2 and Fig. 1). The predictive accuracy with pan-biomarkers for discriminating TA from NTA tissues was 0.747 (*p* = 0.004).

The predictive accuracy of adding clinical features was assessed using regression models. Only age and logPSA are significant with an AUC of 0.631 (*p* = 0.005). We used logPSA to minimize the effect of extreme PSA values. A multiplex model incorporating the pan-biomarkers (6 genes) and clinical information (logPSA and age) identified patients with PC GG ≥ 2 with a high predictive accuracy (AUC 0.815, *p* < 0.0001, Table 3 and Fig. 1).

**Table 3:** Multiplex logistic regression model to detect cancer using panel markers and clinical features (Age, PSA)

Combined	Coefficient	Constant	O.R. (95% CI)	AUC 0.815	*p* value < 0.0001
Age	0.0886	− 0.9658	1.093 (0.996–1.198)		
LPSA	− 2.2394	− 0.9658	0.107 (0.027–0.416)		
C-10 Max	0.0536	− 0.9658	1.055 (0.932–1.194)		
E-1 Max	0.0588	− 0.9658	1.061 (1.001–1.124)		
F-3 Min	− 0.0173	− 0.9658	0.983 (0.920–1.049)		
N-2 Min	− 0.1285	− 0.9658	0.879 (0.752–1.028)		
P-5 Max	0.0497	− 0.9658	1.051 (0.903–1.223)		
S-2 Max	− 0.00503	− 0.9658	0.995 (0.906–1.093)		

*C*, *CAV1*; *E*, *EVX1*; *F*, *FGF1*; *N*, *NCR2*; *P*, *PLA2G16*; *S*, *SPAG4*; *Max*, maximal methylation value between the two biopsies; *Min*, minimal methylation value between the two biopsies; *LPSA*, logPSA; *O.R*., odds ratio; *AUC*, area under the curve value. Number after each letter represents the CG position tested

An alternate statistical analysis using the leave-one-out approach generated a multivariate marker model with the highest AUC of 0.679 (95% CI 0.5868–0.7726). This stepwise selection left only EVX1_CG1 in the model. When age and PSA were included in the final model with EVX1_CG1, the AUC was 0.740 (95% CI 0.6513–0.8287).

Comparing the ability of these markers to differentiate high- (GG ≥ 4) versus low-grade (GG1) cancer was performed in an additional cohort of histologically normal biopsy cores (*n* = 53 and *n* = 52 respectively, [Additional file 1: Table S5]) undergoing prostatectomy. NCR2 alone differentiated high- from low-grade cancer at multiple CGs (Additional file 1: Table S6).

### Methylation assay at multiple biopsies reveals greater heterogeneity across histologically normal prostate tissues in tumor-associated samples than non-tumor samples

**Fig. 2:**
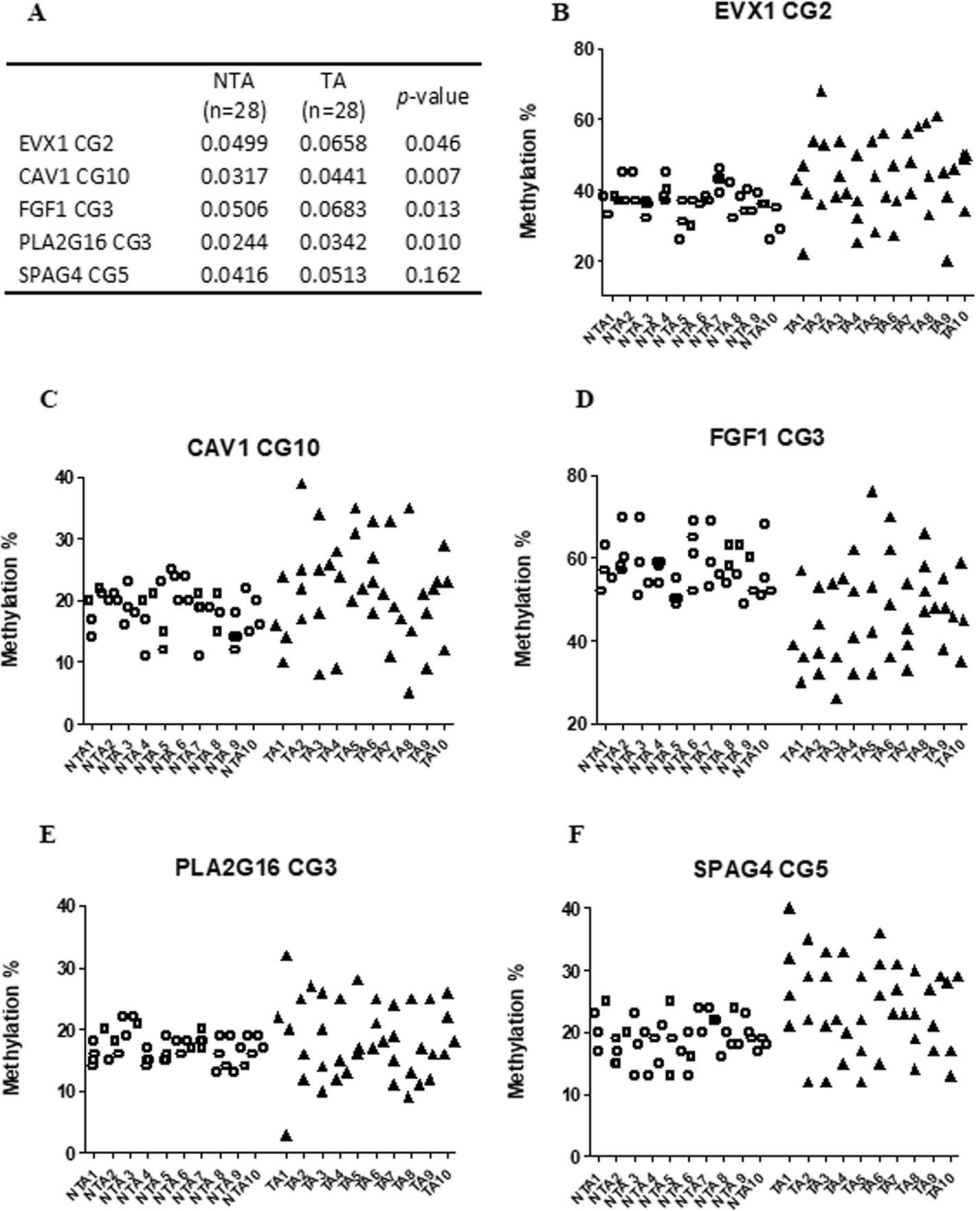
Heterogeneity of methylation between biopsy samples from patients with associated cancer versus those without. Pyrosequencing was performed on biopsy samples as described. **a** Mean value of coefficient of variations from 4 samples for each patient in different cohort. The coefficient of variation (CV) was performed to quantify the variability among each of the patients with four samples using a one-way ANOVA, *p* < 0.05 was considered significantly different between the TA and NTA groups. **b-f** This decreased clustering is noted when additional biopsies (4+) are compared at discrete loci. One CG with the highest predictive accuracy for each gene was selected, ten patients from each group were presented, and the error bar is shown as mean ± SE

To assess the uniformity of the methylation field effect, we compared methylation differences at several loci across benign biopsy blocks. Interestingly, prostate tissues not containing cancer show less variation in methylation at the tested loci between two biopsies (e.g., greater *R* correlation value) than TA samples at the majority (91%) of CGs tested (Additional file 1: Table S7). We expanded this analysis in a subset of 56 subjects with four or more biopsy blocks (28 TA and 28 NTA). Methylation patterns of patients using ≥ 4 biopsies again show more variation in men with cancer than without (Fig. 2a–f). We performed the coefficient of variation (CV) to quantify the variability among each of the patients with four samples using a one-way ANOVA. The CVs of the TA group were significantly higher than the NTA group in *EVX1*, *CAV1*, *FGF1*, and *PLA2G16* (Fig. 2a). Figure 2b–f listed the individual methylation value of every biopsy from each patient. These data indicate that methylation is more heterogeneous in histologically normal prostate tissues associated with tumor (TA) compared with NTA samples.

Given these findings, we questioned whether examining additional biopsy blocks would increase the ability to detect the presence of cancer. In uniplex modeling, of the CpG sites tested, 6 out of 6 *EVX1*, 3/10 *CAV1*, 4/5 *FGF1*, 5/6 *PLA2G16*, and 4/5 *SPAG4* showed improved predictive accuracy (*p* < 0.05, AUCs > 0.6, Table 4). *EVX1*_CG2 alone showed the best predictive value (AUC 0.741, *p* = 0.001). Multiplex models using one CG with the highest AUC per gene increased the AUC to 0.774 (*p* = 0.0004, Table 4).

**Table 4:** Uniplex and multiplex logistic regression model for biomarker performance using an increased number of biopsies (≥ 4 four biopsies)

		Uniplex modeling			
CG	Coefficient	Constant	O.R. (95% CI)	AUC	*p* value
C-3 Max	0.0511	− 1.5444	1.052 (1.002–1.106)	0.611	0.042
C-7 Max	0.0419	− 1.7637	1.043 (1.005–1.083)	0.626	0.028
C-10 Max	0.0924	− 1.8233	1.097 (1.021–1.179)	0.667	0.012
C-10 Mean	0.0992	− 1.6387	1.104 (1.012–1.205)	0.625	0.026
E-1 Mean	0.0937	− 3.6503	1.098 (1.043–1.156)	0.712	0.001
E-1 Max	0.0769	− 3.349	1.080 (1.034–1.128)	0.722	0.001
E-2 Mean	0.1019	− 3.3565	1.107 (1.048–1.170)	0.741	0.001
E-2 Max	0.0807	− 2.9841	1.084 (1.036–1.134)	0.722	0.001
E-3 Mean	0.0667	− 3.1724	1.069 (1.028–1.112)	0.679	0.001
E-3 Max	0.0447	− 2.4064	1.046 (1.017–1.076)	0.660	0.002
E-4 Mean	0.0416	− 3.1792	1.042 (1.004–1.082)	0.654	0.030
E-5 Max	0.0648	− 3.988	1.067 (1.022–1.114)	0.714	0.003
E-5 Mean	0.0692	− 3.8805	1.072 (1.022–1.124)	0.702	0.004
E-6 Max	0.092	− 3.4551	1.096 (1.038–1.158)	0.690	0.001
E-6 Mean	0.1015	− 3.3915	1.107 (1.037–1.181)	0.694	0.002
F-1 Min	-0.0459	3.2474	0.955 (0.915–0.997)	0.623	0.038
F-2 Min	-0.0492	3.2673	0.952 (0.909–0.998)	0.610	0.039
F-3 Min	-0.0597	3.2681	0.942 (0.898–0.988)	0.645	0.015
F-3 Mean	-0.0584	3.5090	0.943 (0.893–0.997)	0.628	0.038
F-4 Min	-0.051	3.6573	0.950 (0.912–0.990)	0.638	0.015
N-2 Mean	-0.1211	4.3103	0.886 (0.764–1.027)	0.586	0.049
P-2 Max	0.0879	− 1.7649	1.092 (1.011–1.179)	0.600	0.025
P-2 Mean	0.1119	− 1.9961	1.118 (1.002–1.248)	0.607	0.045
P-3 Mean	0.1868	− 2.669	1.205 (1.055–1.377)	0.662	0.006
P-3 Max	0.1278	− 2.049	1.136 (1.033–1.250)	0.661	0.009
P-4 Mean	0.0382	− 1.8555	1.039 (1.002–1.077)	0.618	0.038
P-5 Mean	0.1194	− 2.4529	1.127 (1.029–1.234)	0.655	0.010
P-5 Max	0.0961	− 2.1926	1.101 (1.022–1.186)	0.659	0.011
P-6 Mean	0.0694	− 1.6402	1.072 (1.004–1.144)	0.617	0.036
S-1 Max	0.0718	− 1.7891	1.074 (1.014–1.138)	0.630	0.014
S-2 Max	0.0717	− 2.3864	1.074 (1.020–1.132)	0.651	0.007
S-4 Max	0.0741	− 1.6883	1.077 (1.007–1.152)	0.626	0.030
S-5 Mean	0.1648	− 3.1627	1.179 (1.074–1.294)	0.706	0.001
S-5 Max	0.1023	− 2.2471	1.108 (1.040–1.180)	0.681	0.001
		Multiplex modeling			
CpG from each locus				0.774	0.0004
C10 Max	− 0.0176	− 1.9828	0.983 (0.890–1.085)		
E-2 Mean	0.084	− 1.9828	1.088 (1.018–1.162)		
F-3 Min	− 0.031	− 1.9828	0.969 (0.913–1.030)		
N-2 Mean	− 0.0488	− 1.9828	0.952 (0.797–1.139)		
P-3 Mean	0.0339	− 1.9828	1.034 (0.865–1.238)		
S-5 Mean	0.1049	− 1.9828	1.111 (0.977–1.263)		

*C*, *CAV1*; *E*, *EVX1*; *F*, *FGF1*; *N*, *NCR2*; *P*, *PLA2G16*; *S*, *SPAG4; Mean*, mean methylation value of the four biopsies; *Max*, maximal methylation value among the four biopsies, *Min*, minimal methylation value among the four biopsies; *O.R*., odds ratio; *AUC*, area under the curve value. Number after each letter represents the CG position tested

## Discussion

An epigenetic field of cancer susceptibility occurs in aging-related cancers [6, 10] and is especially marked in men with PC [4, 7, 8]. The field effect that arises with and contributes to the development of cancer can be exploited to detect or rule out cancer. The standard approach for PC diagnosis includes histopathological examination of prostate biopsy tissue, but this approach contains a high false-negative rate due to sampling errors. As a result, many men undergo repeated biopsies. Our group has discovered that epigenetic alterations exist not only in the tumor tissue, but also at distance in the histologically benign tissue from patients with PC [4, 9]. Using subjects from multiple institutions, we generated an assay that predicts the presence of PC in histologically benign biopsies. Furthermore, we find that these gene methylation patterns display more heterogeneity in men with cancer elsewhere in the gland than men without suggesting variations in the methylation field effect.

In the current study, *EVX1*, *CAV1*, *PLA2G16*, and *SPAG4* were hypermethylated and *FGF1* and *NCR2* were hypomethylated in TA samples. Each single CG demonstrates robust predictive capabilities. A combination of markers incorporating the six genes allowed for even stronger predictive accuracy (AUC = 0.747). By combining the epigenetic assay with clinicopathological features (PSA, age), the predictive power for PC detection by these field defect markers was improved even more (AUC = 0.815). At each point along the ROC curve, the multiplex model performed better than gene marker or clinical factors alone. Using a cutoff value (PTA of 25%) for the multiple marker combined with PSA and age to detect the presence of cancer yields a 97% sensitivity and 16% specificity, positive predictive value (PPV) of 63%, and negative predictive value (NPV) of 80%.

The current marker analyses indicate that the methylation patterns from patients with cancer are more heterogeneous across the prostate than those found in patients without cancer. This interesting finding gives a window into the biology of the multifocal nature of this disease and proves useful in improving the ability to discriminate risk of associated cancer. Because the methylation patterns in men with associated cancer vary, using maximum or minimum metrics increased the predictive value compared with using the average metric (Additional file 1: Table S2–S4). In determining cervical cancer risk, CpGs exhibiting heterogeneous outlier methylation profiles can improve diagnosis [11]. In breast cancer, DNA methylation outliers in normal breast tissue identify field defects that are enriched in women with cancer [12]. Recent work in prostate suggests that clonal basal stem cells migrate from periurethral ducts [13]. This may give rise to the observed variation in field methylation and contribute to heterogeneity between multifocal PCs.

The current approach employed two biopsy blocks for diagnosis in contrast to other reports that rely on more extensive core analysis (> 5) [14]. Given the finding of this heterogeneity, we increased the number of biopsies analyzed across the prostate gland and find that it improves the assay accuracy in the subset of subjects with this information. In a uniplex model, all CGs demonstrated improved predictive accuracy and increasing AUC values with four or more biopsy blocks compared with two (Table 4). As we obtained four biopsy blocks from 43% of the patients, we did not perform further analyses incorporating this approach with clinical features. Of note, an additional comparison of methylation at these loci between indolent (GG = 1) and aggressive (GG ≥ 3) cancers had to be performed in an independent cohort as all patients in the trial had GG ≥ 2. We observed decreased methylation levels between GG1and GG4/5 (Additional file 1: Table S6) at multiple CGs for the hypomethylated gene NCR2. Further testing on larger cohorts containing a range of cancer grades will be required to evaluate this aspect more definitively.

One factor that makes it difficult to determine an absolute absence of cancer in any cancer detection study and may reflect the lower NPV is that cancer is often difficult to detect even with multiple biopsies as a trial criterion. Our study required at least two negative biopsy sets (24+ cores) to be obtained for entry, and the majority of patients had a negative MRI (62%). Two negative biopsies (without imaging) decrease the risk of missed prostate cancer to less than 9% in previous work [15]. We followed the NTA group over an extended period of time (2+ years) as well. Because PSA is elevated by both cancer and prostate enlargement, and this elevation drives prostate biopsy, the NTA group demonstrates increased size compared with the tumor-associated group (Table 1).

In addition, discrepancies in the way biopsies are obtained between and within institutions might affect methylation values across samples. Biopsies encompassing tissue from the central zone of the prostate, seminal vesicle, or bladder may alter methylation levels due to the inclusion of other tissue types. Heavily inflamed samples were excluded from the current study to avoid this confounding factor. The cell of origin for the methylation changes was not determined by microdissection of the sample since the goal was to evaluate the whole tissue field defect as a marker for cancer presence. Alterations in genomic imprinting of the IGF2 gene, which marks this field defect, appear in the epithelial component [8, 16].

## Conclusions

This field effect improves the detection of PC as demonstrated by application of a methylation assay. Additionally, these abnormalities occur in benign tissue distant from the cancer foci and vary across the normal tissue in the prostate gland. The methylation status of the above biomarkers distinguishes between TA and NTA prostate tissues, marking a field of susceptibility associated with the development of PC.

## Methods

### Tissue samples and histopathology

Individual medical centers obtained institutional review board approval exemption or waiver for the use of archived clinical samples for research purposes. Non-tumor-associated (NTA) control subjects (*n* = 77) had two or more consecutive negative sets of biopsies within 24 or greater months. Tumor-associated (TA) samples were from 52 patients diagnosed with PC who had undergone radical prostatectomy and final pathology was available for grade confirmation. On final pathology, all cancer samples were Gleason Score (GS) ≥3 + 4 = 7 (Grade Group (GG) ≥ 2), considered clinically significant cancer. Other inclusion criteria involved 10–12 total cores per biopsy (separated into distinct regional zones) collected no earlier than 2011, PSA between 3 and 15 ng/mL, and age 50–70 years old. At least two biopsy blocks were requested with each block containing 1–2 biopsy cores and an effort was made to take the normal tissue from the contralateral side away from the detected cancer to avoid contamination. Requested data included ethnicity, family history of PC, positive or negative digital rectal exams, prior negative prostate biopsy, and body mass index. Prostate size was calculated by ultrasound. A total of 176 patients were initially collected, of which 47 (26.7%) were excluded because of the failure to undergo sextant biopsy (*n* = 46) or insufficient biopsy material (*n* = 1) leaving 129 subjects for analysis.

To compare DNA methylation alterations between low GG 1 (GS 3 + 3) and high GG 4/5 (GS ≥ 8), an additional cohort of histologically normal biopsy cores (*n* = 53 and *n* = 52 respectively) undergoing prostatectomy were used. Clinical data comparing high- (GG ≥ 4) versus low-grade (GG1) cancer is provided in Additional file 1: Table S6.

For all specimens, a five-micron section was cut from the non-tumor blocks provided, hematoxylin and eosin (H&E) stained, and centrally reviewed by a fellowship trained genitourinary pathologist (Dr. Wei Huang). Samples with extensive high-grade intraepithelial neoplasia (HGPIN) or atypical small acinar proliferation (ASAP) were excluded.

### Quantitative pyrosequencing

Ten-micron sections were utilized to make DNA from each block. DNA isolation and sodium bisulfite modification were performed according to the manufacturer’s protocol using the EpiTect Plus FFPE Bisulfite Kit (Qiagen, CA, USA). Bisulfite-modified DNA was then amplified using PCR in preparation for pyrosequencing, with either biotinylated forward or reverse primer. All PCR and sequence primers for pyrosequencing were designed using PyroMark Assay Design 2.0 (Qiagen), have been previously described (4). PCR products were captured with streptavidin sepharose beads, denatured to single strand, and annealed to the sequencing primer for the pyrosequencing assay. Human Premixed Calibration Standard with different percentage of methylation (EpigenDx, Hopkinton, MA), human white blood cell DNA, and SssI methylase-treated DNA from human PC cells-Du145 were used as controls in each run. Methylation was quantified with the PyroMark MD Pyrosequencing System (Qiagen) within the linear range of the assay. All samples were analyzed using two independent experiments.

### Statistical analyses

All samples were run in duplicate (two independent experiments) and the two methylation percentage values were averaged. For the cohorts in Table 1, since there are two biopsy tissue blocks from each patient, three metrics (mean, maximum, and minimum) were used to determine significant differences between NTA and TA cohorts. Mean values for each marker were calculated by averaging the methylation of all samples for that cohort. Maximum and minimum values for each marker were calculated by selecting the highest (or lowest) methylation percentage for each patient. At each CpG, a *t* test was performed to analyze the significant differences between NTA and TA groups.

All metrics which significantly differentiated NTA from TA (*p* < 0.05) were entered into a univariate logistic regression model to test their ability to predict the presence of cancer. Area under the curve (AUC) values and *p* values were calculated. The collinearity of individual CpG sites was also assessed using correlation matrices for each gene. Since CG sites correlated highly, only one CG with the highest AUC value for the univariate per locus was selected to enter multivariate logistic regression models to prevent over fitting. The univariate logistic analysis was also performed using clinical factors. Finally, multivariate logistic regression analysis for the performance of biomarkers combined with clinical factors was done. A two-sided *p* value of < 0.05 was considered significant for all hypothesis tests.

A separate statistical approach was used for cross validation of the performance of these biomarkers. The AUCs and the 95% confidence intervals (CI) using DeLong’s method were computed using R 3.4.2 and the pROC package. The CGs with the highest AUC within each gene were selected for further evaluation in a multivariate model of markers, with only one CG per gene included at a time. Stepwise selection was utilized in SAS 9.4. AUC was calculated with 95% CI using the leave-one-out method for validation. The final model included the combination of markers yielding the highest AUC along with age and PSA (with a logarithmic transformation). Drs. Glen Leverson and Kaitlin Woo performed statistical analyses for this manuscript using SAS v.9.4 (SAS Institute, Cary, NC, USA).

## Supplementary information

**Additional file 1: Table S1.** R2 linearity values for methylation pyrosequencing assay at each gene locus. **Table S2.** Mean methylation value (%) with SD for two prostate biopsies. **Table S3.** Maximum methylation value (%) with SD for two biopsies. **Table S4.** Minimum methylation value (%) with SD for two biopsies. **Table S5.** Clinicopathological features of Grade Group = 1 and Grade Group 4/5. **Table S6.** Comparing the ability of the markers to different GG 1 vs GG4/5. **Table S7.** Estimated R correlation between two biopsies.

## Data Availability

All data generated or analyzed during this study are included in this published article and its supplementary information files. *CAV1*, *EVX1*, *FGF2*, *NCR2*, *PLA2G16*, and *SPAG4* sequences are available in GenBank under accession numbers NC_000007.14, NC_000007.14, NC_000005.10, NC_000006.12, NC_000011.10, and NC_000020.11, respectively.

## Abbreviations

APC: Adenomatous polyposis coli
ASAP: Atypical small acinar proliferation
AUC: Area under the curve
CAV1: Caveolin 1
EVX1: Even-skipped homeobox 1
FGF1: Fibroblast growth factor 1
GG: Grade Group
GS: Gleason Score
GSTP1: Glutathione S-transferase pi 1
HGPIN: High-grade intraepithelial neoplasia
NCR2: Natural cytotoxicity triggering receptor 2
NPV: Negative predictive value
NTA: Non-tumor associated
PC: Prostate cancer
PLA2G16: Phospholipase A2 group XVI
PPV: Positive predictive value
PSA: Prostate-specific antigen
PTA: Probability of target attainment
RASSF: Ras association domain family member 1
SPAG4: Sperm-associated antigen 4
TA: Tumor associated

## References

[1] Loeb S, Carter HB, Berndt SI, Ricker W, Schaeffer EM (2011). Complications after prostate biopsy: data from SEER-Medicare. J Urol..

[2] Bennett ST, Wilson AJ, Esposito L, Bouzekri N, Undlien DE, Cucca F (1997). Insulin VNTR allele-specific effect in type 1 diabetes depends on identity of untransmitted paternal allele. The IMDIAB Group. Nat Genet..

[3] Blute ML, Abel EJ, Downs TM, Kelcz F, Jarrard DF (2015). Addressing the need for repeat prostate biopsy: new technology and approaches. Nat Rev Urol..

[4] Yang B, Bhusari S, Kueck J, Weeratunga P, Wagner J, Leverson G (2013). Methylation profiling defines an extensive field defect in histologically normal prostate tissues associated with prostate cancer. Neoplasia..

[5] Wolff EM, Chihara Y, Pan F, Weisenberger DJ, Siegmund KD, Sugano K (2010). Unique DNA methylation patterns distinguish noninvasive and invasive urothelial cancers and establish an epigenetic field defect in premalignant tissue. Cancer Res..

[6] Baba Y, Ishimoto T, Kurashige J, Iwatsuki M, Sakamoto Y, Yoshida N (2016). Epigenetic field cancerization in gastrointestinal cancers. Cancer Lett..

[7] Damaschke NA, Yang B, Bhusari S, Svaren JP, Jarrard DF (2013). Epigenetic susceptibility factors for prostate cancer with aging. Prostate..

[8] Bhusari S, Yang B, Kueck J, Huang W, Jarrard DF (2011). Insulin-like growth factor-2 (IGF2) loss of imprinting marks a field defect within human prostates containing cancer. Prostate..

[9] Truong M, Yang B, Livermore A, Wagner J, Weeratunga P, Huang W (2013). Employing the epigenetic field defect to detect prostate cancer in biopsy-negative patients. The Journal of Urology.

[10] Tahara T, Yamazaki J, Tahara S, Okubo M, Kawamura T, Horiguchi N (2017). Magnifying narrow-band imaging of gastric mucosal morphology predicts the H. pylori-related epigenetic field defect. Sci Rep.

[11] Teschendorff AE, Jones A, Fiegl H, Sargent A, Zhuang JJ, Kitchener HC (2012). Epigenetic variability in cells of normal cytology is associated with the risk of future morphological transformation. Genome Med..

[12] Teschendorff AE, Gao Y, Jones A, Ruebner M, Beckmann MW, Wachter DL (2016). DNA methylation outliers in normal breast tissue identify field defects that are enriched in cancer. Nat Commun..

[13] Moad M, Hannezo E, Buczacki SJ, Wilson L, El-Sherif A, Sims D (2017). Multipotent basal stem cells, maintained in localized proximal niches, support directed long-ranging epithelial flows in human prostates. Cell Rep..

[14] Stewart GD, Van Neste L, Delvenne P, Delrée P, Delga A, McNeill SA (2013). Clinical utility of an epigenetic assay to detect occult prostate cancer in histopathologically negative biopsies: results of the MATLOC study. J Urol..

[15] Roehl KA, Antenor JA, Catalona WJ (2002). Serial biopsy results in prostate cancer screening study. J Urol..

[16] Fu VX, Schwarze SR, Kenowski ML, Leblanc S, Svaren J (2004). Jarrard DF A loss of insulin-like growth factor-2 imprinting is modulated by CCCTC-binding factor down-regulation at senescence in human epithelial cells. J Biol Chem..

