# Supplementary material for: Validation of an epigenetic field of susceptibility to detect significant prostate cancer from non-tumor biopsies
Source: Clin Epigenetics. 2019 Nov 28;11:168. doi: 10.1186/s13148-019-0771-5 (PMC6883627; doi:10.1186/s13148-019-0771-5)
Supplement: Supplementary file 1 — Additional file 1: Table S1. R2 linearity values for methylation pyrosequencing assay at each gene locus. Table S2. Mean methylation value (%) with SD for two prostate biopsies. Table S3. Maximum methylation value (%) with SD for two biopsies. Table S4. Minimum methylation value (%) with SD for two biopsies. Table S5. Clinicopathological features of Grade Group = 1 and Grade Group 4/5. Table S6. Comparing the ability of the markers to different GG 1 vs GG4/5. Table S7. Estimated R correlation between two biopsies. [file 13148_2019_771_MOESM1_ESM.pdf]

Table S1 R^2 linearity values for methylation pyrosequencing assay at each gene locus

	CG1	CG2	CG3	CG4	CG5	CG6	CG7	CG8	CG9	CG10
CAV1	0.9931	0.9947	0.9924	0.9871	0.9961	0.9738	0.9854	0.9979	0.9968	0.9923
EVX1	0.9956	0.9945	0.9968	0.9892	0.9929	0.992				
FGF1	0.9941	0.9922	0.9845	0.9843	0.9862					
NCR2	0.9957	0.9941	0.9961							
PLA2G16	0.9891	0.9922	0.978	0.9874	0.981	0.9945				
SPAG4	0.9946	0.9935	0.997	0.9896	0.9885					

Human DNA standards with different percentages of methylation (EpigenDx) were used to test the precision of pyrosequencing. R2 values were generated by plotting the theoriotical and measured values.

Table S2 Mean methylation value (%) with SD for two prostate biopsies

		CG1	CG2	CG3	CG4	CG5	CG6	CG7	CG8	CG9	CG10
CAV1	NTA	10.7(3.6)	25.0(6.5)	32.0(8.1)	21.8(6.3)	22.9(6.7)	23.5(6.5)	42.9(9.6)	12.3(3.4)	27.6(4.6)	19.3(4.9)
	TA	11.0(3.2)	25.4(6.1)	33.8(7.3)	23.1(6.3)	23.3(6.4)	25.5(7.2)	45.5(9.6)	13.1(3.4)	28.4(5.3)	21.2(5.1)*
EVX1	NTA	40.2(8.7)	34.2(9.4)	48.8(9.4)	82.8(10.1)	58.8(10.2)	35.3(6.0)				
	TA	45.5(9.2)*	38.8(8.9)*	55.4(12.6)*	86.5(13.0)*	62.8(11.0)*	38.9(9.2)*				
FGF1	NTA	68.7(8.0)	64.6(6.4)	55.4(6.9)	70.3(8.2)	71.1(7.0)					
	TA	67.7(8.3)	63.3(7.2)	53.0(8.2)	68.5(8.8)	71.3(7.9)					
NCR2	NTA	64.7(4.2)	33.8(2.3)	65.4(3.4)							
	TA	64.5(4.0)	32.9(2.7)	65.3(3.9)							
PLA2G16	NTA	39.3(7.3)	20.1(3.5)	54.0(11.6)	54.0(11.6)	22.5(4.5)	26.9(5.6)				
	TA	42.2(7.6)*	21.6(4.2)*	59.5(12.6)*	59.5(12.6)*	24.6(4.7)*	29.7(6.7)*				
SPAG4	NTA	25.3(6.4)	32.8(7.5)	30.7(6.0)	23.3(4.2)	19.4(4.5)					
	TA	26.8(6.2)	34.8(7.6)	31.6(6.2)	24.8(5.4)	22.2(5.4)*					

*T-TEST, $p<0.05$.

Table S3 Maximum methylation value (%) with SD for two biopsies

		CG1	CG2	CG3	CG4	CG5	CG6	CG7	CG8	CG9	CG10
CAV1	NTA	11.8(4.5)	26.9(7.3)	33.9(8.6)	23.8(7.3)	24.5(7.4)	26.0(8.2)	45.9(10.8)	13.3(4.0)	29.4(5.6)	21.0(6.1)
	TA	12.4(3.9)	27.9(7.1)	36.6(8.3)	25.8(7.4)	25.3(6.8)	29.1(9.2)	50.5(11.6)*	14.4(3.8)	30.7(6.2)	23.3(6.0)*
EVX1	NTA	42.2(9.6)	36.5(10.1)	52.1(11.4)	87.1(11.9)	61.5(10.8)	37.3(6.7)				
	TA	49.5(10.4)*	42.3(9.3)*	61.7(15.4)*	91.7(12.4)*	67.0(10.8)*	41.9(10.8)*				
FGF1	NTA	70.9(7.7)	66.6(6.5)	57.5(7.1)	72.5(8.1)	74.0(7.6)					
	TA	71.0(9.6)	66.4(8.1)	56.4(9.2)	72.2(9.5)	74.7(8.2)					
NCR2	NTA	66.1(4.7)	34.7(2.6)	66.7(3.7)							
	TA	66.5(5.1)	34.1(3.1)	66.9(4.2)							
PLA2G16	NTA	42.3(8.6)	21.4(4.3)	16.2(3.6)	58.3(14.2)	23.8(4.7)	28.6(6.5)				
	TA	45.9(9.0)*	23.7(4.7)*	18.2(4.4)*	65.5(16.1)*	26.6(5.3)*	32.3(7.9)*				
SPAG4	NTA	26.8(6.4)	34.6(7.5)	33.0(7.8)	25.1(4.8)	21.0(5.2)					
	TA	29.3(6.7)*	37.9(8.2)*	34.5(7.8)	26.9(5.6)	24.8(6.8)*					

*T-TEST, $p<0.05$.

Table S4 Minimum methylation value (%) with SD for two biopsies

		CG1	CG2	CG3	CG4	CG5	CG6	CG7	CG8	CG9	CG10
CAV1	NTA	9.6(3.2)	23.1(6.5)	30.1(8.4)	19.8(6.1)	21.1(6.6)	21.0(6.1)	39.9(9.7)	11.3(3.2)	25.8(4.7)	17.8(4.5)
	TA	9.6(3.2)	22.9(6.2)	30.9(7.8)	20.5(6.7)	21.3(7.0)	22.0(7.2)	40.5(10.0)	11.8(3.7)	26.2(5.6)	19.1(5.4)
EVX1	NTA	38.2(8.8)	32.0(9.7)	45.5(9.1)	78.4(10.9)	56.1(10.8)	33.4(6.4)				
	TA	41.5(10.5)	35.3(10.1)	49.1(13.8)	81.3(17.1)	58.6(13.1)	35.9(9.5)				
FGF1	NTA	66.4(8.9)	62.6(7.0)	53.3(7.6)	68.1(9.0)	68.2(8.3)					
	TA	64.4(9.0)	60.2(8.6)	49.6(9.0)*	64.9(10.3)	67.9(9.4)					
NCR2	NTA	63.4(4.6)	32.8(2.6)	64.2(3.9)							
	TA	62.6(3.9)	31.7(3.0)*	63.7(4.6)							
PLA2G16	NTA	36.3(7.8)	18.8(3.7)	14.1(3.1)	49.7(11.6)	21.2(4.7)	25.1(5.5)				
	TA	38.5(8.9)	19.4(4.7)	15.0(4.1)	53.5(12.6)	22.6(5.2)	27.1(7.1)				
SPAG4	NTA	23.8(6.8)	30.9(8.1)	28.3(5.7)	21.6(4.8)	17.7(5.0)					
	TA	24.3(7.0)	31.7(8.5)	28.7(6.5)	22.8(6.1)	19.6(5.5)*					

*T-TEST, $p<0.05$.

Table S5 Clinicopathological features of Grade Group=1 and Grade Group 4/5

	Grade Group =1	Grade Goup 4/5	Total	*p*-value
Patients, n	53	52	105	---
Age (yr)	59 (48-75)	60.6 (44-72)	59.8 (44-75)	0.22
PSA (ng/mL)*	5.6 (0.4-14.7)	10.8 (1.3-41.2)	8.2 (0.4-41.2)	<0.01
PSA Density (ng/mL)*	0.124 (0.01-0.66)	0.231 (0.03-1.02)	0.18 (0.01-1.02)	<0.01
Prostate Size (g)	49.9 (21.7-98.2)	49.9 (15.3-118.4)	49.9 (15.3-118.4)	0.99
BMI (kg/m^3)	28.4 (20.5-39)	29.5 (22.5-44)	29 (20.5-44)	0.22
Ethnicity:				
Caucasian	100% [53/53]	88.5% [46/52]	94.3% [99/105]	---
Family History:*				
Positive	39.6% [21/53]	26.9% [14/52]	33.3% [35/105]	---
DRE:*				
Positive	5.9% [1/17]	47.4% [9/19]	27.8% [10/36]	---
Pathological Stage:				
T2	51	29	80	---
T2a	12	4	16	---
T2b	1	1	2	---
T2c	38	24	62	---
T3a	2	11	13	---
T3b	0	12	12	---

*Some samples are missing data; Prostate specific antigen (PSA). Body mass index (BMI). All data represented as mean (range) unless otherwise specified

Table S6 Comparing the ability of the markers to different GG 1 vs GG4/5

	**CG1**	**CG2**	**CG3**	**CG4**	**CG5**	**CG6**	**CG7**	**CG8**	**CG9**	**CG10**
**CAV1**										
GG1	15	34	40	28	29	33	56	16	34	27
GG4/5	15	34	43	31	31	34	59	18	35	28
T-TEST	0.79	0.82	0.24	0.40	0.33	0.6	0.4	0.37	0.66	0.8
**EVX1**										
GG1	48	42	65	93	64	38				
GG4/5	49	43	68	94	66	41				
TTEST	0.91	0.99	0.58	0.95	0.57	0.04				
**FGF1**										
GG1	63	61	47	67	73					
GG4/5	63	60	48	67	71					
T-TEST	0.89	0.74	0.85	0.76	0.48					
**NCR2**										
GG1	72	36	70							
GG4/5	71	34	69							
T-TEST	**0.018**	**0.047**	0.219							
**PLA2G16**										
GG1	49	26	20	82	31	37				
GG4/5	46	25	21	77	29	38				
T-TEST	0.17	0.62	0.74	0.31	0.21	0.001				
**SPAG4**										
GG1	33	41	39	29	28					
GG4/5	32	41	37	29	27					
T-TEST	0.59	0.71	0.43	0.63	0.48					

Table S7 Estimated R correlation between two biopsies

		CG1	CG2	CG3	CG4	CG5	CG6	CG7	CG8	CG9	CG10
CAV1	NTA	0.148	0.224	0.398	0.289	0.359	0.146	0.220	0.445	0.010	0.348
	TA	0.284	0.279	0.273	0.219	0.182	-0.002	0.170	0.000	-0.043	0.159
EVX1	NTA	0.444	0.412	0.201	0.286	0.453	0.337				
	TA	0.226	0.305	0.106	-0.027	0.106	0.265				
FGF1	NTA	0.465	0.298	0.444	0.429	0.102					
	TA	0.099	-0.030	0.195	0.089	0.061					
NCR2	NTA	0.309	0.111	0.192							
	TA	0.086	-0.070	0.095							
PLA2G16	NTA	0.126	0.348	0.237	0.097	0.286	0.028				
	TA	0.052	-0.083	-0.026	0.118	0.181	0.000				
SPAG4	NTA	0.419	0.361	0.175	0.282	0.184					
	TA	0.139	0.162	0.067	0.222	0.090					
